# Systems Biology and Pangenome of *Salmonella* O-Antigens

**DOI:** 10.1128/mBio.01247-19

**Published:** 2019-08-27

**Authors:** Yara Seif, Jonathan M. Monk, Henrique Machado, Erol Kavvas, Bernhard O. Palsson

**Affiliations:** a Department of Bioengineering, University of California, San Diego, La Jolla, California, USA; b Department of Pediatrics, University of California, San Diego, La Jolla, California, USA; Korea Advanced Institute of Science and Technology

**Keywords:** *Salmonella*, genome analysis, metabolism, serogroups

## Abstract

O-antigens are glycopolymers in lipopolysaccharides expressed on the cell surface of Gram-negative bacteria. Variability in the O-antigen structure constitutes the basis for the establishment of the serotyping schema. We pursued a two-pronged approach to define the basis for O-antigen structural diversity. First, we developed a bottom-up systems biology approach to O-antigen metabolism by building a reconstruction of *Salmonella* O-antigen biosynthesis and used it to (i) update 410 existing *Salmonella* strain-specific metabolic models, (ii) predict a strain’s serogroup and its O-antigen glycan synthesis capability (yielding 98% agreement with experimental data), and (iii) extend our workflow to more than 1,400 Gram-negative strains. Second, we used a top-down pangenome analysis to elucidate the genetic basis for intraserogroup O-antigen structural variations. We assembled a database of O-antigen gene islands from over 11,000 sequenced *Salmonella* strains, revealing (i) that gene duplication, pseudogene formation, gene deletion, and bacteriophage insertion elements occur ubiquitously across serogroups; (ii) novel serotypes in the group O:4 B2 variant, as well as an additional genotype variant for group O:4, and (iii) two novel O-antigen gene islands in understudied subspecies. We thus comprehensively defined the genetic basis for O-antigen diversity.

## Abstract (precis)

Lipopolysaccharides are a major component of the outer membrane in Gram-negative bacteria. They are composed of a conserved lipid structure that is embedded in the outer leaflet of the outer membrane and a polysaccharide known as the O-antigen. O-antigens are highly variable in structure across strains of a species and are crucial to a bacterium’s interactions with its environment. They constitute the first line of defense against both the immune system and bacteriophage infections and have been shown to mediate antimicrobial resistance. The significance of our research is in identifying the metabolic and genetic differences within and across O-antigen groups in *Salmonella* strains. Our effort constitutes a first step toward characterizing the O-antigen metabolic network across Gram-negative organisms and a comprehensive overview of genetic variations in *Salmonella*.

## INTRODUCTION

O-antigens are made up of glycan repeat units which contain a combination of two to eight sugar residues. Variations in the final structure occur as a result of the order in which the glycans are linked, the type of linkage between residues, and the type of linkage between O units. These glycans are frequently O-antigen specific, and their biosynthesis is well studied in *Salmonella*, *Escherichia*, and *Shigella* (1, 2). There are 47 known O-antigen structures and 21 different O glycans in *Salmonella* (2). The Kauffmann-White-Le Minor serotyping scheme is based on the combination of O and H antigens expressed at the surface (3). Serogroups are defined based on the characterized O-antigen structure alone and are subdivided into serovars based on the H-antigen structure. A differentiating feature across *Salmonella* serogroups is the first sugar found in the O-antigen unit, which can either be an *N*-acetylglucosamine/*N*-acetylgalactosamine or a galactose residue. *Salmonella* strains falling under the latter category are the most frequently isolated and typed (2).

Most O-antigen biosynthetic genes are colocated in a genomic island known as the *rfb* cluster, where sequence diversity faithfully reflects the diversity of O-antigen structures. The island has dissimilar G+C content with respect to the rest of the genome, reflecting the fact that it is a hot spot for homologous recombination and horizontal gene transfer (4, 5). Currently, the sequences for 47 *rfb* clusters corresponding to each *Salmonella* serogroup have been identified, a subset of which are also found in Escherichia coli strains. The *rfb* cluster is a genomic locus subject to high selection pressure as is evidenced by the extensive variability of lipopolysaccharide (LPS) structures across *Salmonella* strains. Although genetic variability within serogroups has been reported in a few cases, no study has systematically analyzed this diversity across all serogroups. In addition, due to their genetic diversity, the O-antigen and LPS metabolic subsystems have long presented a challenge when propagating metabolic networks across strains using comparative genomics (6–8). Understanding the consequences of O-antigen sequence diversity constitutes a step forward to understanding the diversity in host-pathogen and host-phage interactions.

Here, we both assemble an O-antigen gene island pangenome and reconstruct the metabolic pathways for O-antigen biosynthesis incorporating them with a pangenome reconstruction of *Salmonella* metabolism (8). Genome-scale models of metabolism (GEMs) offer a powerful way to quantitatively analyze the energy and carbon demands of metabolite synthesis. We add here a much-needed functionality to the *Salmonella* GEM, which can also be extended to other organisms, i.e., the prediction of a strain’s serogroup and its ability to synthesize different LPS and O-antigen structures. Taking advantage of the structural similarity of O-antigens across different organisms, we show that these pathways can be exported to other Gram-negative strains.

## RESULTS

### O-antigen biosynthetic pathways reveal unexpected nonglycolytic extracellular nutrient sources.

**FIG 1:**
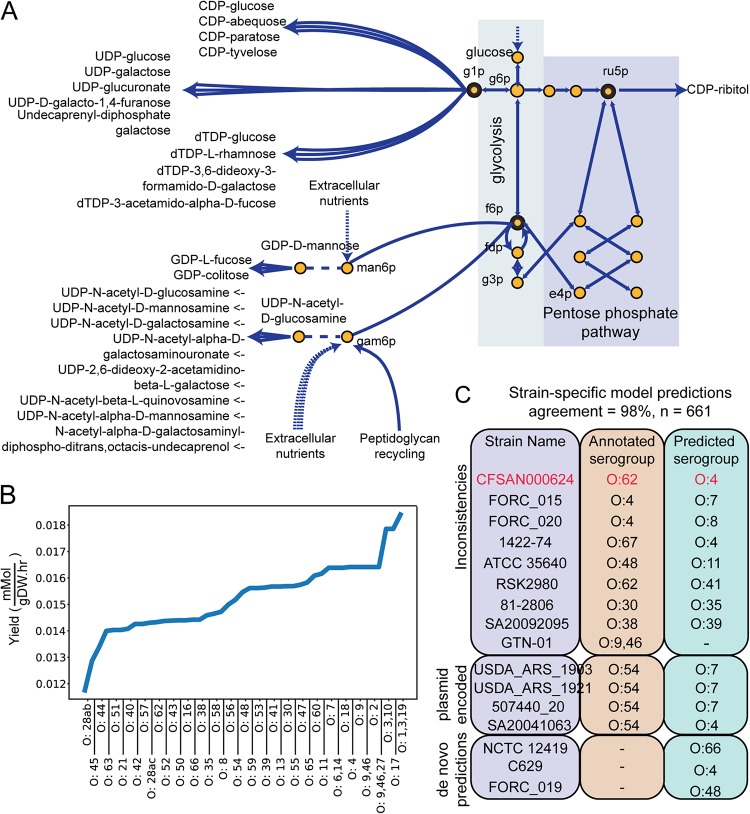
Metabolic modeling of O-antigen biosynthesis. (A) O-antigen glycan biosynthetic pathways included in Oag.v1 and pan-STM.v1.2. (B) Yield [mmol of LPS/g (dry weight)/h] for the various O-antigen structures with growth rate set at 80% of wild-type growth rate. (C) Inconsistencies between the systems biology serogroup predictions and the strains’ annotations. The systems biology workflow achieved a 98% accuracy. We highlight here the cases for which we observed (i) inconsistencies resulting from observed genotype, (ii) inconsistencies likely resulting from plasmid encoded O-antigen genes but for which we did not have access to plasmidic sequences, and (iii) *de novo* serogroup predictions. Strain CFSAN000624 was serologically tested experimentally, and the outcome matched the model-driven prediction (i.e., it is serogroup O:4).

To date, only a subset of O-antigen precursor biosynthetic pathways have been formally described through computational models (and are available in BiGG and other metabolic databases [9]), with LPS_O:4_ (group O:4) being the only lipopolysaccharide structure for which a full metabolic pathway description exists in *Salmonella.* However, there are 46 known serogroups in *Salmonella*, each characterized by a unique O-antigen structure, LPS structure, and *rfb* cluster (with the exception of serogroup O:28, which is subdivided into subserogroups O:28ab and O:28ac). To complete the O-antigen metabolic subsystem, we curated and reconstructed the biosynthetic pathways for 46 out of 47 O-antigen structures (Fig. 1A; see also Table S1 in the supplemental material). For this purpose, we conducted an extensive literature review and followed an established reconstruction protocol (10). This endeavor uncovered gaps of knowledge in O-antigen metabolism, specifically that (i) the substrate specificity and pathway order of glycosyltransferases and acetyltransferases and that (ii) the O-antigen precursor biosynthetic pathways for group O:61 have not been fully characterized. The resulting O-antigen metabolic model (Oag.v1) yielded 348 genes, 695 reactions, and 590 metabolites.

10.1128/mBio.01247-19.1 TABLE S1 Metabolic reconstruction for O-antigen biosynthesis. This file includes gene, reaction, and metabolite information, as well as predicted LPS-specific gene and reaction essentiality. Download Table S1, CSV file, 0.4 MB. Copyright © 2019 Seif et al. 2019 Seif et al. This content is distributed under the terms of the Creative Commons Attribution 4.0 International license.

We subsequently updated the pangenomic reconstruction of *Salmonella* pan-STM.v1.1 (which contains all known and modeled *Salmonella* metabolic pathways) (8) with the content from Oag.v1. As a result, the content of pan-STM.v1.2 was 23% larger and encompassed 1,718 genes and 3,372 reactions. We observed that glucose-1-phosphate, ribulose-5-phosphate, and fructose-6-phosphate serve as common precursors to the biosynthesis of different subsets of O-antigen glycans, with CDP-ribitol being the only glycan capable of being synthesized from ribulose-5-phosphate. In addition, various extracellular nutrients which bypass the reliance on glycolytic flux can serve as precursors to O-antigen glycan biosynthesis. These include d-mannose, d-mannose-6-phosphate, d-gluconate, d-glucosamine, and d-glucosamine-6-phosphate, as well as products obtained from anhydro muropeptide recycling (which serve to recycle peptidoglycan). To assess the relative carbon yield of each LPS structure, we fixed the growth rate at 80% of wild-type growth on glucose plus M9 minimal medium (see Materials and Methods and Fig. 1B). The predicted yield varied as much as 56.5% across structures, with the LPS structure for serogroup O:28ab having the lowest yield and serogroup O:1,3,19 having the highest. These results reflect the stoichiometric differences between metabolic pathways. We proceeded to computationally simulate gene essentiality for the production of each LPS structure and found that, on average, 39 ± 2.57 genes become essential when LPS is included in the biomass objective function (11) (see Table S1 in the supplemental material).

### Pan-STM.v1.2 accurately predicts the serogroup of *Salmonella* strains.

To validate our reconstruction, we used a data set of 663 genomic sequences covering a range of 28 serogroups (Table S2). Of these, 410 are complete (gapless) and have existing metabolic reconstructions (12), and 253 are incomplete (more than one contig) but are experimentally serotyped and sequenced by the same group (13). We reconstructed or updated strain-specific networks for all 665 strains using the approach highlighted by Seif et al. (8). We proceeded to predict each strain’s capability to synthesize any of the 46 O-antigen structures using flux balance analysis (see Materials and Methods).

10.1128/mBio.01247-19.2 TABLE S2 Feature table for 663 strain-specific reconstructions. The annotated and predicted serogroup is listed for each strain. The ability of each strain to synthesize all O-antigen structures is also listed. The strain-specific models are available in sbml format on BioModels under accession MODEL1907100002. Download Table S2, CSV file, 1.3 MB. Copyright © 2019 Seif et al. 2019 Seif et al. This content is distributed under the terms of the Creative Commons Attribution 4.0 International license.

As a result, serogroup predictions for 647 of 659 were accurate, amounting to an overall agreement of 98% (4 strains were not serotyped). Because of the similarity in biosynthetic pathways, strains of serogroups O:9 and O:3,10 were predicted to be able to synthesize two O-antigen structures each (structures O:9 and O:2 and structures O:1,3,19 and O:3,10, respectively). Interestingly, strain NCTC 12419 lacked wzz_ST_, which is involved in regulating the O-antigen polymer length and could not synthesize long O-antigen polymers (14). There were four instances of misclassified strains that were annotated as serogroup O:54. *rfb*_O54_ is plasmid encoded and has been previously reported for serovar Borreze, which also harbors an inactivated chromosomal O-antigen locus (15). All four strains carried a genomic *rfb* cluster and the strain-specific models predicted that three strains (annotated as serovar Montevideo) could synthesize O:7, whereas the latter strain could synthesize O:4 (annotated as serovar Borreze). However, none of the plasmid sequences were available for these strains, and we could not verify O:54 biosynthesis capability. Strain GTN-01 (serogroup O:9,46) was predicted to be incapable of synthesizing any O-antigen structure. Upon further inspection, we noticed that the *rfb* cluster was missing eight genes essential for O-antigen metabolism.

For the remaining eight inconsistencies, we could not find any of the biosynthetic genes corresponding to the annotated serogroup in the O-antigen gene island. Instead, we found the intact *rfb* cluster matching that of the predicted serogroup. For example, two strains, FORC_015 and FORC_020 (annotated as serogroup O:4), were predicted to synthesize LPS_O8_ and LPS_O6,7_ and carried complete *rfb*_O8_ and *rfb*_O6,7_ clusters, respectively. However, they both lacked all of the *rfb*_O4_-specific genes. It is possible that *rfb*_O4_ was plasmid encoded in these strains; however, we did not have access to the corresponding plasmid sequences. Similar cases were observed for strains CFSAN000624, ATCC 35640, RSK2980, 81-2806, SA20092095, and 1422-74 (Fig. 1C). We serologically tested strain CFSAN000624 experimentally, and the results agreed with the model-driven group O:4 prediction (Table 1).

**TABLE 1:** Experimental serotyping assays outcome

Strain	Test type^*a*^	Outcome^*b*^			
		Polyvalent 42-67 (81061), *Salmonella*	Monovalent O:4,5 (23839), *Salmonella*	Monovalent O:15 (48243), E. coli	Polyvalent A-S+Vi (73519), *Salmonella*
E. coli K-12 MG1655	NC	N	N	N	N
S. enterica strains					
subsp. *diarizonae* CFSAN044910	Test	P	N	N	N
Serovar Stanleyville CFSAN000624	Test	N	P	N	P
Serovar Enteritidis EC20100101	PC	N	N	N	P
Serovar Enteritidis SA20094177	PC	N	N	N	P
Serovar Bovismorbificans 3114	PC	N	N	N	P

a PC, positive control; NC, negative control.

b P, presence of seroagglutination; N, absence of seroagglutination.

A total of four strains were not annotated for a serogroup in our data set. Our strain-specific model approach predicted (i) S. bongori NCTC 12419 as serogroup O:66, (ii) S. enterica strain C629 as serogroup O:4, and (iii) S. enterica strain FORC_019 as serogroup O:9. We could not assign a serogroup for the fourth strain, however, because of a single gene deletion of *nnaD* (an *O*-acetyltransferase) from the O:48 gene island. We hypothesize that the strain (Salmonella bongori strain N268-08) synthesizes a modified O:48 antigen structure with a nonacetylated l-fucosamine residue. In general, these results show that our metabolic-model-based approach achieves high predictive accuracy while enabling a mechanistic interpretation of model predictions.

### O-antigen glycan biosynthesis is variably shared across several Gram-negative species.

**FIG 2:**
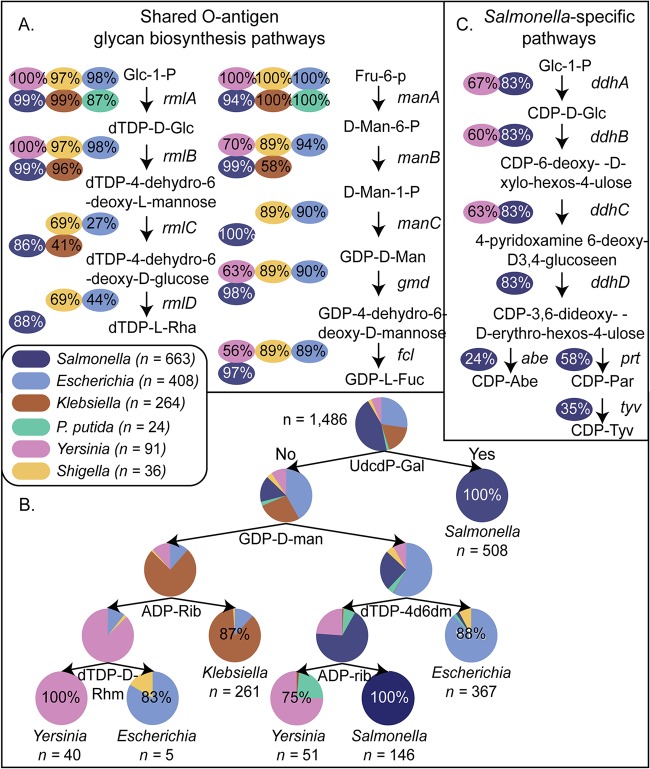
O-antigen glycan biosynthesis capability across Gram-negative genera. (A) Conservation of O-glycan biosynthesis genes across genera. (B) Predicted capability to synthesize O-antigen glycans across various genera: *Escherichia*, *Salmonella*, *Yersinia*, *Klebsiella*, *Pseudomonas*, and *Shigella*. The most conserved feature is the ability to synthesize UDP-*N*-acetylglucosamine and UDP-glucose. (C) *Salmonella*-specific O-glycan metabolic pathway. Abbreviations: Glc-1-*P*, glucose-1-phosphate; dTDP-d-Glc, dTDP-d-glucose; dTDP-l-Rha, dTDP-l-rhamnose; Fru-6-*P*, fructose-6-phosphate; d-Man-6-*P*, d-mannose-6-phosphate; d-Man-1-*P*, d-mannose-1-phosphate; GDP-d-Man, GDP-d-mannose; GDP-l-Fuc, GDP-l-fucose; Glc-1-*P*, glucose-1-phosphate; CDP-Abe, CDP-abequose; CDP-Par, CDP-paratose; CDP-Tyv, CDP-tyvelose; UdcdP-gal, undecaprenyl diphospho-galactose; dTDP-4d6dm, dTDP-4-dehydro-6-deoxy-l-mannose; ADP-rib, ADP-ribose; CDP-Glc, CDP-glucose.

O-antigen biosynthesis is shared across Gram-negative bacteria and can be acquired through cross-species horizontal gene transfer (5, 16). We thus extended our workflow to other Gram-negative genera for which highly curated GEMs exist, including *Escherichia* (*n = *408), *Klebsiella* (*n *=* *264), *Yersinia* (*n *=* *91), and *Shigella* (*n *=* *36) spp. and Pseudomonas putida (*n = *24). We considered strains for which complete genomic sequences are available on PATRIC (12) and built strain-specific draft models of metabolism. Upon simulating O-antigen and O-antigen precursor biosynthesis, we found that only two glycolipids are commonly synthesized across all strains: UDP-*N-*acetyl*-* d-glucosamine and UDP-glucose (see Table S3 in the supplemental material). The former also serves as a precursor to peptidoglycan biosynthesis, while the latter is a precursor to colanic acid and capsular polysaccharide biosynthesis. Similarly, the biosynthesis of dTDP-d-glucose, dTDP-4-dehydro-6-deoxy-d-glucose, UDP-*N*-acetyl-d-mannosamine, and UDP-galactose was conserved in more than 97% of the strains. About 21% of E. coli strains synthesized UndPP-GalNAc, whereas only 8.3% of *Shigella* and 1.5% of *Salmonella* strains could. Eleven E. coli strains were predicted to completely synthesize and assemble *Salmonella* O-antigens, including serotypes O:35 (strains 95JB1, 95NR1, 11128, and 268-78-1), O:58 (strain DSM 103246), and O:6,14 (strains 042, C4, CFSAN061770, and UMN026). Most glycolipids (e.g., GDP-colitose, dTDP-3-acetamido-α-d-fucose, and dTDP-3,6-dideoxy-3-formamido-d-galactose) could only be synthesized by a reduced number of strains probably due to their specificity to the O-antigen metabolic subsystem and to a subset of O-antigen structures. Two biosynthetic genetic pathways—(i) *rmlABCD* and (ii) *manAB*, *gmd*, and *fcl*—were highly shared across genera (Fig. 2A) .

10.1128/mBio.01247-19.3 TABLE S3 Feature table for 1,486 Gram-negative strains with the capability of each strain to synthesize O-antigen structures and precursors. Download Table S3, CSV file, 0.9 MB. Copyright © 2019 Seif et al. 2019 Seif et al. This content is distributed under the terms of the Creative Commons Attribution 4.0 International license.

The most discriminating capability was an ability to synthesize UndPP-galactose, ADP-ribose, dTDP-4-dehydro-6-deoxy-l-mannose, GDP-d-mannose, and dTDP-d-rhamnose (Fig. 2B). ADP-ribose biosynthesis was highly conserved across *Klebsiella* strains (261 of 264), and GDP-d-mannose biosynthesis was shared across 19 of 24 P. putida strains. Only *Salmonella* strains could produce UndPP-galactose (which is the first sugar in the O-antigen unit of multiple clinically relevant *Salmonella* serovars), indicating that this capability may have developed across *Salmonella* strains after species diversification. In addition, *Shigella* and *Escherichia* strains were not distinguishable, suggesting common ancestry in their O-antigen biosynthetic module. Finally, seven glycolipids were *Salmonella* specific: CDP-abequose, CDP-paratose, CDP-tyvelose, UDP-*N*-acetyl-d-galactosamine, UDP-*N*-acetyl-α-d-galactosaminouronate, dTDP-3-acetamido-α-d-fucose, and dTDP-3,6-dideoxy-3-formamido-d-galactose (Fig. 2C).

### Extending the knowledge base to over 11,000 *Salmonella* strains.

**FIG 3:**
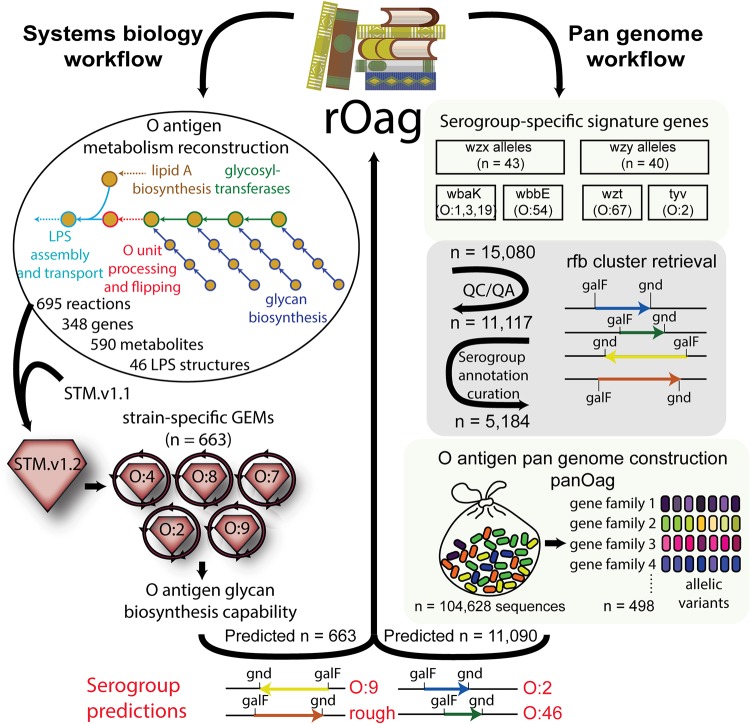
O-antigen gene island database construction and modeling workflow chart. O-antigen gene islands were retrieved from the PATRIC database and quality checked (48). An O-antigen pangenome was constructed by pooling together the O-antigen island gene content across strains. Genes were clustered into gene families by means of sequence homology using CD-HIT (45), and reference gene clusters were chosen for all 46 serogroups. Genes were then annotated with a metabolic function and used to reconstruct an O-antigen metabolic network. Serogroup annotations were derived from the metadata associated with each genome, and serogroup predictions were made using serogroup-specific signature orthologous groups.

Our systems biology approach is a bottom-up approach that is highly accurate, reliable, and encompasses many attractive features, such as the power to predict a strain’s O glycan and the identification of the active metabolic pathways feeding into O-antigen biosynthesis in any user-defined nutritional environment and genetic background. However, this approach relies on high-quality genomic sequences to reach its full predictive power at the strain level. We designed an alternative approach that bypasses this requirement to assess the full genetic diversity across *Salmonella rfb* clusters. Making use of our curated and nonredundant reconstructed metabolic network, we selected one representative *rfb* cluster for each serogroup and assembled them into the “O-antigen reference database” or “rOag” (Fig. 3; see also Table S4 in the supplemental material). We linked our curated annotations to all genes across rOag, resulting in a total of 372 annotated genes.

10.1128/mBio.01247-19.4 TABLE S4 Representative O-antigen gene clusters feature tables and protein sequences for 46 *Salmonella* serogroups. Each sequence is annotated with a gene family ID as identified by CD-Hit. The gene families used to predict each strain’s serogroup are also marked. Download Table S4, CSV file, 0.3 MB. Copyright © 2019 Seif et al. 2019 Seif et al. This content is distributed under the terms of the Creative Commons Attribution 4.0 International license.

Next, we selected a subset of signature genes per serogroup which fully distinguish the 46 serogroups (including O-antigen polymerases, *wzy* and flippases, and *wzx* [see Materials and Methods]). We proceeded to retrieve and quality check O-antigen gene islands for a total of 11,117 *Salmonella* genomes from PATRIC and curated the metadata available to obtain the strains’ serogroup. We found the necessary information to associate a serogroup to 5,184 strains (46.2%). We constructed the O-antigen pangenome (PanOag) by pooling together all 104,628 O-antigen genes and clustered them into 498 orthologous groups (see the supplemental material). Using the selected signature genes, we predicted the serogroups for all strains and obtained 98.5% agreement. To benchmark our approach, we ran SeqSero on our data set (13), which yielded a 98.1% agreement (Table S5). There were 34 strains for which prediction discrepancies between SeqSero and PanOag occurred. PanOag and SeqSero made no predictions for 15 strains each. The failure cases for PanOag are discussed below and may constitute a pitfall of this approach. However, 14 of the 15 cases in which SeqSero made no predictions were predicted correctly by PanOag.

10.1128/mBio.01247-19.5 TABLE S5 Feature table for the 11,117 *Salmonella* strains. The metadata for all strains is enriched with the serogroup annotation extracted from the strain’s annotations (see methods), as well as the serogroup predictions obtained from the signature gene family search. Observed genetic variations such as gene duplications, deletions, gene decays, and insertions is indicated for each strain. For all genetic variations, the loci affected (e.g., deleted genes and inserted genes) are indicated by their annotations (when they are available) or by the gene family ID (i.e., Cluster ID). The accession number for the contig on which the O-antigen gene island is located, along with start and end positions are also listed for each strain. Download Table S5, CSV file, 2.3 MB. Copyright © 2019 Seif et al. 2019 Seif et al. This content is distributed under the terms of the Creative Commons Attribution 4.0 International license.

### Gene duplication, decay, and deletion contribute to intraserogroup genetic diversity and lead to loss of function.

Serogroup predictions and annotations matched well under the signature genes approach. However, when we attempted to predict a strain’s serogroup by matching the full gene content between O-antigen islands, our accuracy dropped to 94.2%. This drop was due to variations in the gene content between O-antigen islands of the same serogroup. While gene order was highly conserved, 3.2% of *Salmonella* strains were affected by either gene decay and/or gene deletion. Tandem gene duplication occurred in 706 *rfb* clusters, with serogroups O:4 and O:2 exhibiting the highest frequency of duplications. The most commonly duplicated genes in serogroup O:2 were UTP–glucose-1-phosphate uridylyltransferase and mannosyltransferase. Gene duplication events constitute one of the many mechanisms that drive the acquisition of new gene functions (17) and can lead to elevated intraspecific gene expression variation (18).

**FIG 4:**
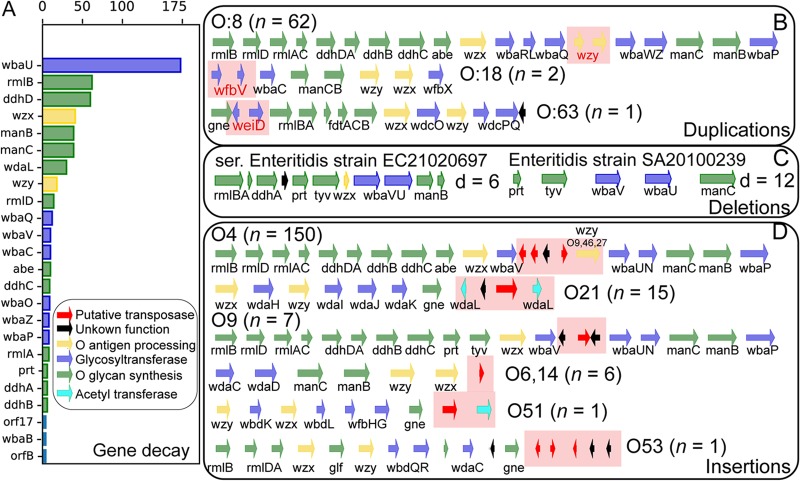
Sources of genetic variability in the content of *rfb* clusters. (A) Gene decay across orthologous groups. (B) Tandem gene duplications. (C) Gene deletions (d = no. genes missing with respect to the reference *rfb* cluster). In these cases, the strains likely exhibit a rough phenotype (e.g., they do not synthesize O-antigens). (D) Gene insertions. Note that only one sample of each serogroup affected by insertions is shown but that the *rfb* cluster may vary in content across the *n* strains (due to additional insertions, deletions, or duplications). Similarly, we only show a subset of serogroups and *rfb* clusters with gene duplication and deletion events because there are too many serogroups which are affected by these phenomena (see Table S7 for a full list).

Another evolutionary route after gene duplication is the decay of one of the two copies. We found a total of 1,189 decaying genes across 612 strains (see Materials and Methods and Table S5 in the supplemental material), with the most highly represented orthologous groups being *wbaU* (*n *=* *182), *rmlB* (*n* = 62), *ddhD* (*n* = 60), *wzx* (*n* = 41), *manB* (*n* = 39), and *manC* (*n* = 39) (Fig. 4A and B). The frequency of occurrence of at least one gene decay across serogroups ranged between 3.1% (group O:18) and 100% (group O:2). Of these, 458 genes (across 392 strains) both were duplicated and exhibited gene decay in at least one copy; of these, 65 exhibited gene decay in both copies. In the latter case, the gene is likely nonfunctional and may affect the strain’s ability to synthesize O-antigen macromolecules. Gene deletion affected 175 strains across 20 serogroups, in which case one of the O-antigen genes was completely missing from the O-antigen gene island. The most extreme cases featured strains exhibiting 12 (SA20100239), 9 (CFSAN000197), and 6 (EC21020697) gene deletions in their O-antigen gene island (Fig. 4C). Notable, *wzy* was the only gene found between *galF* and *gnd* in strain CFSAN000197 (serovar Bareilly). The most commonly missing functions were *wbaL* (*n* = 74), *wbaN* (*n* = 40), and *wbaU* (*n* = 36). We hypothesize that the corresponding strains exhibit the rough-type phenotype, in which the lipopolysaccharides produced and embedded in the outer membrane only consist of lipid A and core oligosaccharide but lack an O-antigen.

10.1128/mBio.01247-19.7 TABLE S7 Strains for which no predictions could be made. JSpecies results for species identification of strains with high frequency of novel genetic material (*n* > ). Download Table S7, XLSX file, 0.1 MB. Copyright © 2019 Seif et al. 2019 Seif et al. This content is distributed under the terms of the Creative Commons Attribution 4.0 International license.

### Of the orthologous groups in PanOag, 25.3% fall outside the orthologous groups in rOag.

We found that 341 strains carried a gene family (orthologous group) in their *rfb* cluster that was absent from the reference *rfb* cluster for that serogroup. The majority of unexpected orthologous groups (77.8%) were not annotated with a catalytic activity. In total, 126 orthologous groups were not present in any of the reference clusters and were searched against the EggNOG database for a putative functional annotation (19). We assigned general metabolic categories to 74 orthologous groups; 22 of these were glycosyltransferases, 15 were transposases, and another 15 were annotated with a non-O-antigen-related metabolic function (Table S6). The rest of the orthologous groups (*n* = 42) could not be assigned to any function or general metabolic category. We observed that insertion elements (including transposases and insertion sequences) appeared in the O-antigen loci of 184 strains spanning seven serogroups and often resulted in the disruption of genes involved in O-antigen biosynthesis such as acetyltransferases and glycosyltransferases (Fig. 4D). For example, all Minnesota strains (*n* = 16, serogroup O:21) carried an insertion element disrupting *wdaL*, an acetyltransferase. Transposons flanking genes that have yet to be annotated with a catalytic activity were distributed across other serogroups as well and may be the genetic basis for unknown/novel serovar variants.

10.1128/mBio.01247-19.6 TABLE S6 Orthology predictions and functional annotations of gene families falling outside the curated O-antigen pangenome. All genes that were successfully annotated by EggNOG are featured in this table along with the cluster ID (i.e., gene family ID). Download Table S6, CSV file, 0.03 MB. Copyright © 2019 Seif et al. 2019 Seif et al. This content is distributed under the terms of the Creative Commons Attribution 4.0 International license.

### PanOag uncovers the group O:4 B2 variant in novel serotypes, as well as an additional genotype variant for group O:4.

Of the 341 strains, 184 carried at least one insertion element, with 150 of these predicted to be serogroup O:4. Transposon insertions in group O:4 occurred predominantly in the *wbaV*-*wbaU* region. All Schwarzengrund strains (*n* = 103, serogroup O:4) carried three transposases, a 391-amino-acid oligosaccharide repeat unit polymerase (*wzy*), and a putative protein that contained only 46 amino acids. O-antigen polymerases catalyze the stacking of O-antigen units (made up of three to eight glycans) onto a lipid carrier in the periplasm, generating specific linkages between the O units. Polymerases vary highly in sequence across serogroups because of their specificity to the O unit and linkage type. The polymerase sequence found across Schwarzengrund strains was homologous to *wzy_O9_*_,_*_46_*_,_*_27_* (that catalyzes an α1-6 linkage type instead of the α1-2 linkage type). Strains of Bredeney (all 23), Altendorf (1), Cerro (1 of 15), Eko (all 3), and Heidelberg (103 of 104) also carried *wzy_O9_*_,_*_46_*_,_*_27_* flanked by two transposases, which were absent across all other 3,865 strains of serogroup O:4. The group O:4 O-antigen polymerase is located outside the *rfb* cluster and shares no similarity with the polymerase identified here. We searched all of the strains carrying *wzy_O9_*_,_*_46_*_,_*_27_* for *wzy_O:4_* and found it to be present in all cases. Reeves et al. made a similar observation across strains of serovars Schwarzengrund, Schleissheim, Sloterdijk, and Vellore, all of which were classified as group B2 variants and have the O27 epitope (20). Epitope O27 has been identified across strains of Bredeney and Altendorf (3), and the presence of *wzy_O9_*_,_*_46_*_,_*_27_* is likely the corresponding genetic basis. However, the O27 epitope has yet to be reported across strains of Cerro, Eko, and Heidelberg, despite *wzy_O9_*_,_*_46_*_,_*_27_* being carried in the majority of Heidelberg strains (except one). In addition to *wzy_O9_*_,_*_46_*_,_*_27_*, all three Eko strains carried the O:3,10 acetyltransferase (*wbaK*) downstream of *wbaP* (21), suggesting that their O-antigen structure may be acetylated. This constitutes yet another group O:4 genetic variant, here named B3.

### PanOag distinguishes non-*Salmonella* strains and uncovers two new *Salmonella* gene islands.

We could not assign a serogroup annotation to 24 *rfb* clusters using the signature gene-based approach. Of these, 15 clusters were missing *wzx* and/or *wzy*, and many exhibited multigene deletion events, with the most extreme cases being the *rfb* clusters for (i) strain CFSAN000197 (serovar Bareilly), which contained only one gene (*wzx*) in its O-antigen island instead of the expected 7 genes, and (ii) strain 818 (serovar Cerro), with three genes deleted (*wzy*, *wbaB*, and *wbaD*) in addition to an acetyltransferase (*wfbX*) insertion. Closer examination of the remaining eight *rfb* clusters revealed that they contained multiple orthologous groups that fell outside rOag. To confirm that the genomic sequences really corresponded to *Salmonella* strains, we calculated their tetranucleotide signature correlation against the JSpecies internal reference database (22) and found that only two of the eight strains were predicted to belong to the *Salmonella* genus while the other six were identified as either *Citrobacter* species (*n* = 5) or *Raoultella* species (*n* = 1) (Table S7). Disagreement between species-level classification by routine laboratory methods and classification by genomic similarity has been previously observed to occur at a frequency of 12% (23). In light of these results, we discarded the six sequences from further analysis.

**FIG 5:**
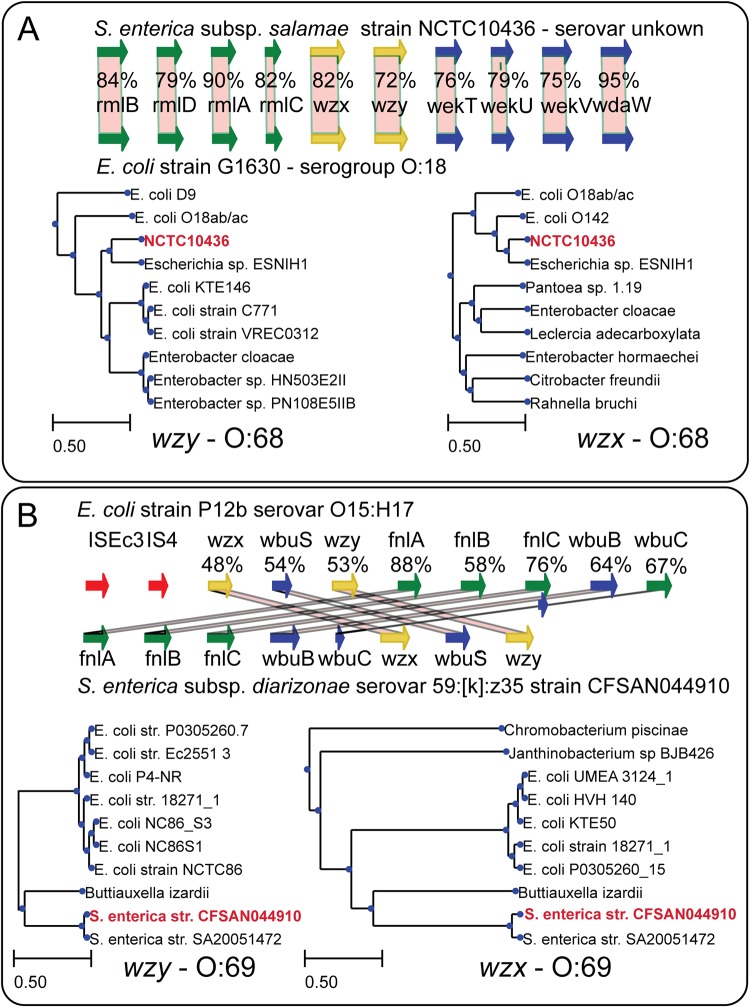
Two novel *Salmonella* O-antigen gene island genotypes, O:68 and O:69, were uncovered in the *rfb* pangenome. Three strains were found to carry novel O-antigen gene clusters. (A) Orthologs for strain NCTC10436 were found in the *rfb* cluster for *E. coli* strain G1630 of serogroup O:18ab (24, 49), with the closest matches for *wzx* and *wzy* identified in strain ESNIH1. (B) The gene content between strains CFSAN044910 and SA20051472 was similar to E. coli P12b, but the order was not conserved. The closest matches for *wzx* and *wzy* were identified in Buttiauxella izardii.

The three remaining strains were NCTC10436 (subsp. *salamae*, serogroup O:44), strain CFSAN044910 and strain SA20051472 (subsp. *diarizonae*, serogroup O:59) (Table S8). Strain NCTC10436 contained 10 genes in its cluster, 5 of which did not fall under any of the annotated orthologous groups, including novel variants of *wzx* and *wzy*, and three novel glycosyltransferases. A BLAST search against the nonredundant NCBI database identified close orthologs carried by E. coli strain ESNIH1. However, ESNIH1 did not have an annotated serogroup. We thus screened all 10 genes against the E. coli O-antigen database assembled by DebRoy et al. using pBLAST and found matches for all 10 genes in E. coli strain G1630 (group O:18ab, Fig. 5A) (24). Interestingly, the percentage of sequence homology was highest at the edges and lowest toward the center.

10.1128/mBio.01247-19.8 TABLE S8 Feature table for the O-antigen gene island of strains CFSAN055910 and SA20051472 (subsp. *diarizonae*) and NCTC10436 (subsp. *salamae*). Download Table S8, XLSX file, 0.01 MB. Copyright © 2019 Seif et al. 2019 Seif et al. This content is distributed under the terms of the Creative Commons Attribution 4.0 International license.

The *rfb* cluster for strains CFSAN044910 and SA20051472 contained eight genes, with only three falling outside the O-antigen knowledge base, including new variants of *wzx*, *wzy*, and *wbuS*. We found orthologs for all genes in the E. coli strain P12b O-antigen gene island, annotated as serovar O15:H17 (Fig. 5B). However, the sequence of the genes was not conserved. Instead, the order of two syntenic blocks (*wzx-wbuS-wzy* and *fnlA-fnlB-fnlC-wbuB-wbuC*) was flipped, with a higher percentage sequence identity shared with *fnlA-fnlB-fnlC-wbuB-wbuC* than with *wzx-wbuS-wzy*, suggesting that these two blocks were acquired at different times. We named these two genotypes O:68 and O:69, respectively. We acquired and tested strain CFSAN044910 for seroagglutination with E. coli O:15 antisera and *Salmonella* polyvalent O-antiserum OMF (Table 1). Seroagglutination occurred upon O-antiserum OMF exposure but not upon O:15 antiserum exposure. We searched the entire genomic sequences of CFSAN044910 and SA20051472 but found no matches for *wdcF*, *wdcG*, *wdaZ*, *wzx_O59_*, or *wzy_O59_*. We conclude that O:69 was not expressed and that a second O-antigen island could be present in an unsequenced area of the genome or plasmid.

## DISCUSSION

This study represents the first comprehensive attempt to elucidate the systems biology of O-antigen biosynthesis in Gram-negative bacteria, including the construction of a knowledge base and associated computational models. We use the knowledge base to predict the strain-specific capability to produce various O-antigen structures and O-antigen precursors in *Salmonella* and more than 1,400 Gram-negative strains, and as a reference database to exhaustively analyze the genetic diversity in O-antigen gene islands across *Salmonella* strains.

Genome-scale metabolic model-driven serogroup predictions covering 665 *Salmonella* strains yields 98% agreement. Failure cases in which strains carry both a genomic and a plasmid-borne *rfb* cluster highlight one of the pitfalls of *in silico* approaches. We successfully validate experimentally one of the eight remaining inconsistencies, with the model-driven prediction proving to be correct. The addition of the O-antigen metabolic subsystem yields a 23% increase in metabolic gene content in pan-STM.v1.1 (*Salmonella* pan reactome), nearly doubling the size of the accessory reactome of *Salmonella* (*n* = 382 reactions that are not shared across all *Salmonella* strains) (8). This result indicates that the *Salmonella* accessory reactome currently underrepresents the full metabolic diversity of the species and may still increase further in size when additional network reconstruction efforts are undertaken. Furthermore, this effort highlights the fact that metabolic reconstructions should no longer be restricted to a single strain of a species. Instead, to assess the full metabolic capabilities of a species, reconstruction endeavors should proceed at the species level, one metabolic subsystem at a time. In addition, despite being a well-studied species, our reconstruction efforts reveal knowledge gaps in *Salmonella* O-antigen biosynthesis. Finally, merging O-antigen biosynthesis to central metabolism will allow for the application of strain design approaches and serve as a mechanistic tool for designing glycoconjugate-based *Salmonella* vaccines (25).

Because the O-antigen metabolic network is converted into a mathematical format, it is easily transferable to available genome-scale reconstructions (regardless of the species). Notably, not only is O-antigen biosynthesis shared across Gram-negative bacteria, but the biosynthetic gene clusters are also often horizontally transferred across species. We constructed strain-specific metabolic models enriched with the *Salmonella* O-antigen biosynthetic pathways across five other species and simulated both O-antigen and O-antigen precursor biosynthesis capability. Unsurprisingly, a subset of E. coli strains share the ability to synthesize multiple *Salmonella*-specific O-antigens. The results demonstrate that O-antigen precursor biosynthesis capability can differentiate some Gram-negative species and shed some light onto the evolutionary trajectory of O-antigen biosynthesis, indicating that some O-antigen pathways existed before species diversification, while others evolved or were acquired later. Given the ubiquity of O-antigen pathways, we argue that O-antigen metabolism should be treated as a panspecies metabolic module.

To conduct a comprehensive study of the genetic diversity of *Salmonella* O-antigen gene islands we used a comparative genomics approach. We assembled a database of O-antigen gene islands, identified serogroup-specific orthologous groups, and predicted the serogroup for more than 11,000 strains of *Salmonella* (achieving an agreement with experimental metadata of 98.2%). The *rfb* cluster database reveals extensive intraserogroup genetic modifications, with evidence of both added and/or subtracted O-antigen metabolic functions with respect to known metabolic pathways. We observed novel functions (such as acetyltransferases) in strains across multiple serogroups (including serogroup B2) and loss of function due to pseudogene formation, gene deletion, or gene disruption by insertion elements. Diversification of O-antigen structures can arise from phage lysogenization (evidenced by insertion and transposon elements disrupting open reading frames in the O-antigen gene island or by the carriage of glycosyltransferase operons outside the *rfb* cluster causing phase variation) (3, 26). This phenomenon results in the induction of bacteriophage resistance in *E. coli* and *Salmonella* strains (27, 28). Alternatively, loss of function can affect the strains’ ability to synthesize an O-antigen. Although O-antigen expression is not always necessary for a strain’s survival, it does affect the strain’s ability to survive in some niches (29, 30). For example, rough *Salmonella* mutants lose the ability to penetrate the lymph nodes, spleen, and liver and instead colonize the intestinal tract, as opposed to their isogenic counterpart (31). In total, tandem gene duplication, pseudogene formation, deletion, and transposon/prophage-mediated insertion affect the genetic composition of 994 *rfb* cluster spanning 27 serogroups.

We identify two novel *Salmonella* O-antigen genotypes in S. enterica subsp. *salamae* strain NCTC10436 and S. enterica subsp. *diarizonae* strain CFSAN044910. Strains of subspecies *diarizonae* and *salamae* are less often isolated and sequenced than strains of subspecies *enterica*, suggesting that there may be other O-antigen genotypes in these and other understudied *Salmonella* subspecies that have yet to be identified. Both genotypes are found across E. coli strains, suggesting a common ancestry for the O-antigen gene islands between the two species. However, follow-up experiments reveal that one of the two genotypes may not be expressed, suggesting that the gene island is inactivated, as in serogroup O:54 (32).

Altogether, our results constitute the most comprehensive pangenome analysis of *Salmonella* O-antigen gene islands, which comes as a result of combining the bottom-up metabolic reconstruction approach with the top-down pangenome approach. The resulting reconstructions and databases can be used to further study different aspects of O-antigen metabolism evolution and genotype-phenotype associations and can be combined with additional factors contributing to structural variations of O-antigens, such as the insertion of glycosyltransferase operons.

## MATERIALS AND METHODS

### Construction of a *Salmonella* O-antigen biosynthesis knowledge base.

Reference *rfb* clusters were obtained from various literature sources and online databases, with the priority given to those strains which were serotyped experimentally, curated with gene annotations and sequenced by the same authors. The selected reference *rfb* clusters were further curated against the reported *rfb* clusters by Liu et al. (2). We henceforth refer to this collection of curated gene islands as the reference O-antigen gene cluster (rOag). We obtained sequences for 46 of 47 complete *rfb* clusters but only found the sequences for *wbaV* and *wzy* for serogroup O:9,46,27 (33). For each serogroup, we reconstructed a full O-antigen biosynthetic pathway complete with LPS assembly and transport. While most O-antigen-specific biosynthetic genes are located within these clusters, some are present elsewhere in the genome. We used literature review to locate and identify these genes and complete the corresponding metabolic pathways. When the glycosyltransferase substrates were not known, the O-unit assembly was lumped into one reaction. The biosynthetic pathway for one of the glycans for group O:61 is not yet fully characterized and was therefore left out. Two different pathways were built for O:28ab and O:28ac (which are categorized as the same serogroup) due to significant variation in O-antigen structure and corresponding genetic island. As part of the reconstruction process, we corrected the annotation for 182 genes (*tyv*), which are actually pseudogenes. The repeat unit for O:2 and O:9 are similar save for the presence of tyvelose in O:9, which is replaced by paratose in O:2 due to a frameshift mutation in *tyv* (CDP-paratose 2-epimerase).

The degree of O-antigen polymerization is regulated by a combination of genes (*wzzB*, *wzzST*, and *wzzVL*) (14, 34). We therefore reconstructed three pathways for each O-antigen structure to accommodate an average of three observed polymer length distributions: short (15 O units), long (25 O units), and very long (100 O units). The chemical formula for each O polymer and downstream metabolite was calculated using mass balance analysis. We included all biosynthetic pathways in one metabolic model separately (Oag.v1), and updated the pangenome metabolic model of *Salmonella* (pan-STM.v1.1) with the O-antigen biosynthesis module (pan-STM.v1.2). We adjusted the chemical formulae for 21 metabolites to correct for the 21 reactions which were not mass balanced in the benchmark model. Metabolite charges were computed using ChemAxon (35), setting the modeled pH at 7.1. We used the COBRApy package to complete all of the steps described above (36). To ensure that all pathways were complete, we simulated the capability of pan-STM.v1.2 to produce each LPS structure iteratively by setting the lower bound to the biomass function at 80% of wild-type growth on M9 plus glucose minimal medium and the optimization objective LPS export to 1. For the synthesis of each LPS structure, we ran flux-balance analysis using the model.optimize() functionality of the COBRApy package to compute the relative yield of each LPS structure (Fig. 4B), as well as the cobra.flux_analysis.find_essential_genes function to simulate gene essentiality.

### Predicting the LPS and O-glycan biosynthesis capability across *Salmonella* strains.

We used three datasets to validate and test the O-antigen biosynthesis model: (i) a data set of incomplete genomic sequences for which laboratory serovar annotation exists, (ii) a previously published data set (8) consisting of 410 *Salmonella* strain-specific models built from an analysis of complete genomic sequences of *Salmonella*, and (iii) 1,285 complete genomic sequences for Gram-negative strains retrieved from PATRIC and quality checked. We retrieved genome-scale models of metabolism (GEMs) for E. coli (iML1515) (37), *Salmonella* (pan-STM_v1.2) (8), *Yersinia* (iPC815) (38), Pseudomonas putida (iJN1411) (39), and *Klebsiella* (iYL1228) (40) strains and updated them with pathways from STM_Oag. We subsequently built strain-specific models according to a similar workflow, as previously reported (8). Briefly, homologous genes were identified via bidirectional best BLAST hit between each strain and modeled genes in the corresponding reference GEM. When a gene was not found but was essential for a reaction (e.g., did not have a modeled homolog), the reaction was removed. Gene presence or absence was thus used to tailor the reconstructed networks to each strain. The genomic sequences for the first data set was quality checked for the completeness of the *rfb* cluster, while those of the third data set were quality checked by excluding strains with a high ratio of unidentified nucleotide bases (>0.5%). In this analysis we did not exclude plasmids from homology searches. In fact, we screened all of the *Salmonella* plasmid sequences (*n = *376) available on PATRIC (12) for O-antigen biosynthesis genes, but we found no matches. The biomass function of each reference model was updated to exclude O-antigen-specific metabolites (such as glycolipids). After we adjusted the networks to each strain’s genetic background, the models were gap filled such that growth on M9 plus glucose minimal medium could be achieved. The process of gap filling consists of adding the minimum number reactions necessary to achieve growth (41). LPS and O-antigen glycan biosynthesis capability was tested by iteratively adding a sink reaction for each of the 46 LPS structures and O-antigen glycans, setting the objective function to 1, and simulating for growth (by running flux balance analysis [42]). Yield values exceeding 0.001 mmol/g (dry weight)/h were considered to indicate that a strain is able to synthesize the corresponding glycolipid.

### Experimental serotyping assays.

Strains CFSAN044910 and CFSAN000624 were acquired from the Center for Food Safety and Applied Nutrition (43, 44). Serotyping was performed by seroagglutination using commercial antisera (SSI Diagnostica, Denmark) and following the supplier’s instructions. Briefly, strains were streaked onto tryptic soy agar medium from –80°C stocks and grown overnight at 37°C. A single colony was mixed with a drop (20 μl) of antiserum for each different serum. Agglutination observed within the first 10 s after mixing was considered a positive result. *E. coli* K-12 MG1655 was used as a negative control because it has the rough-type phenotype. *S. enterica* serovar Enteritidis strain EC20100101, *S. enterica* serovar Enteritidis strain SA20094177, and *S. enterica* serovar Bovismorbificans strain 3114 were used as positive controls. See Table 1 for the serotyping assay results.

### Construction of an O-antigen pangenome database and *rfb* sequence analysis.

We parsed the PATRIC database (12) to extract the *rfb* clusters cross strains spanning the *Salmonella* genus. For this purpose, we searched for the two signature genes (UTP–glucose-1-phosphate uridylyltransferase and 6-phosphogluconate dehydrogenase) known to flank the O-antigen cluster by using a keyword search of open reading frame annotated functions. Genomic regions were selected when both keywords were located on the same contig, with fewer than 35 coding DNA sequences separating them. We searched a total of 15,080 genomic sequences, which yielded 11,117 *rfb* clusters. Next, we assembled all sequences into a pan-O-antigen gene island database (PanOag) and proceeded to identify the set of nonredundant orthologous genes by using CD-HIT (minimum sequence identity of 0.9) (45). Briefly, CD-HIT clusters gene sequences into orthologous groups when sequence homology meets a certain threshold. Once serogroup predictions were made, we proceeded to compare the gene family content of each *rfb* cluster with the expected gene content for that serogroup (obtained from the curated reference genomic islands) for deletion, duplication, and insertion events. To identify decaying genes, we started by assuming that all genes annotated in the Oag.ve and rOag are functional. Then, for each annotated gene, we searched for the sequences clustered in the same gene family with an amino acid sequence length lower than 50% of the curated reference. When a gene family was not annotated as part of the reference *rfb* annotation step, it was both searched against the nonredundant NCBI protein database using BLASTp and run through the EggNOG functional annotation platform using the HMMER option (19).

### Serogroup prediction scheme.

We proceeded to identify serogroup-specific signature orthologous groups from rOag. For our starting point, we chose the O-antigen flippase (*wzx*) and O-antigen polymerase (*wzy*) as signature orthologous groups because their genetic sequence is highly variable across serogroups as a result of their specificity to the O-antigen structure (13). In fact, they generally formed distinct orthologous groups for each serogroup. This approach alone worked to distinguish most serogroups except for O:54, O:2, O:4, O:9, O:9,46,37, O:9,46, O:1,3,19, O:3,10, and O:67. To distinguish O:2 from O:9 strains, we identified the strains that harbor an L2A substitution in the CDP-paratose 2-epimerase (*tyv*) amino acid sequence which is specific to group O:2 (46). Similarly, *wbaK* served to distinguish serogroup O:3,10 from O:1,3,19. Most *Salmonella* O-antigens are synthesized via the Wzy-dependent pathway in which an O unit is assembled in the cytoplasm, flipped across the periplasmic membrane via Wzx, polymerized in the periplasm via Wzy, and finally transported to the outer membrane. In contrast, the O-antigen biosynthetic pathway for group O:67 is ATP-binding cassette transporter dependent and therefore does involve Wzy or Wzx (2, 47). In this pathway, polymerization occurs in the cytoplasm, and the heteropolymer is subsequently transported across the membrane. As such, we chose *wzt* as the signature gene family for group O:67. Similarly, the O:54 O polysaccharide is a homopolymer which Keenleyside and Whitfield reported as being likely transported while being polymerized (15). We chose *wbbE* as the signature gene family for group O:54. We proceeded to compare our predictions with experimental observations. The serogroup was extracted from each strain’s metadata either through the strain’s name, its serotype, its antigenic formula, or its annotated serogroup (when available). To map strain names and serotypes to a serogroup, we used the White-Kauffman-Le Minor scheme reported by Institut Pasteur (3). We found sufficient metadata to assign a serogroup for 5,170 strains. After comparing our predictions with the annotations, we noticed that a subset of strains of serogroup O:4 could initially not be distinguished from strains of serogroup O:9,46,27 because a subset of strains also carried *wzy_O9_*_,_*_46_*_,_*_27_*. We subsequently added *abe* as an additional signature gene family for group O:4, as well as a B2 and putative B3, O:68 and O:69 representative gene island in rOag. We chose strain NCTC6026 (serovar Bredeney) as a B2 representative because its O-antigen island gene content was the most common across B2 strains and because its genomic sequence is complete.

## References

[1] Liu B, Knirel YA, Feng L, Perepelov AV, Senchenkova SN, Wang Q, Reeves PR, Wang L 2008 Structure and genetics of *Shigella* O antigens. FEMS Microbiol Rev 32:627–653. doi:10.1111/j.1574-6976.2008.00114.x. 18422615

[2] Liu B, Knirel YA, Feng L, Perepelov AV, Senchenkova SN, Reeves PR, Wang L 2014 Structural diversity in *Salmonella* O antigens and its genetic basis. FEMS Microbiol Rev 38:56–89. doi:10.1111/1574-6976.12034. 23848592

[3] Grimont PAD, Weill F-X, Others. 2007 Antigenic formulae of the Salmonella serovars. WHO collaborating centre for reference and research on Salmonella 9.

[4] Wang L, Reeves PR 2000 The *Escherichia coli* O111 and *Salmonella enterica* O35 gene clusters: gene clusters encoding the same colitose-containing O antigen are highly conserved. J Bacteriol 182:5256–5261. doi:10.1128/jb.182.18.5256-5261.2000. 10960113 PMC94677

[5] Hester SE, Park J, Goodfield LL, Feaga HA, Preston A, Harvill ET 2013 Horizontally acquired divergent O-antigen contributes to escape from cross-immunity in the classical bordetellae. BMC Evol Biol 13:209. doi:10.1186/1471-2148-13-209. 24067113 PMC3849452

[6] Monk JM, Charusanti P, Aziz RK, Lerman JA, Premyodhin N, Orth JD, Feist AM, Palsson BØ 2013 Genome-scale metabolic reconstructions of multiple *Escherichia coli* strains highlight strain-specific adaptations to nutritional environments. Proc Natl Acad Sci U S A 110:20338–20343. doi:10.1073/pnas.1307797110. 24277855 PMC3864276

[7] Magnúsdóttir S, Heinken A, Kutt L, Ravcheev DA, Bauer E, Noronha A, Greenhalgh K, Jäger C, Baginska J, Wilmes P, Fleming RMT, Thiele I 2017 Generation of genome-scale metabolic reconstructions for 773 members of the human gut microbiota. Nat Biotechnol 35:81–89. doi:10.1038/nbt.3703. 27893703

[8] Seif Y, Kavvas E, Lachance J-C, Yurkovich JT, Nuccio S-P, Fang X, Catoiu E, Raffatellu M, Palsson BO, Monk JM 2018 Genome-scale metabolic reconstructions of multiple Salmonella strains reveal serovar-specific metabolic traits. Nat Commun 9:3771. doi:10.1038/s41467-018-06112-5. 30218022 PMC6138749

[9] King ZA, Lu J, Dräger A, Miller P, Federowicz S, Lerman JA, Ebrahim A, Palsson BO, Lewis NE 2016 BiGG models: a platform for integrating, standardizing and sharing genome-scale models. Nucleic Acids Res 44:D515–D522. doi:10.1093/nar/gkv1049. 26476456 PMC4702785

[10] Thiele I, Palsson BØ 2010 A protocol for generating a high-quality genome-scale metabolic reconstruction. Nat Protoc 5:93–121. doi:10.1038/nprot.2009.203. 20057383 PMC3125167

[11] Feist AM, Palsson BO 2010 The biomass objective function. Curr Opin Microbiol 13:344–349. doi:10.1016/j.mib.2010.03.003. 20430689 PMC2912156

[12] Wattam AR, Abraham D, Dalay O, Disz TL, Driscoll T, Gabbard JL, Gillespie JJ, Gough R, Hix D, Kenyon R, Machi D, Mao C, Nordberg EK, Olson R, Overbeek R, Pusch GD, Shukla M, Schulman J, Stevens RL, Sullivan DE, Vonstein V, Warren A, Will R, Wilson MJC, Yoo HS, Zhang C, Zhang Y, Sobral BW 2014 PATRIC, the bacterial bioinformatics database and analysis resource. Nucleic Acids Res 42:D581–D591. doi:10.1093/nar/gkt1099. 24225323 PMC3965095

[13] Zhang S, Yin Y, Jones MB, Zhang Z, Deatherage Kaiser BL, Dinsmore BA, Fitzgerald C, Fields PI, Deng X 2015 *Salmonella* serotype determination utilizing high-throughput genome sequencing data. J Clin Microbiol 53:1685–1692. doi:10.1128/JCM.00323-15. 25762776 PMC4400759

[14] Murray GL, Attridge SR, Morona R 2003 Regulation of *Salmonella* Typhimurium lipopolysaccharide O antigen chain length is required for virulence; identification of FepE as a second Wzz. Mol Microbiol 47:1395–1406. doi:10.1046/j.1365-2958.2003.03383.x. 12603743

[15] Keenleyside WJ, Whitfield C 1996 A novel pathway for O-polysaccharide biosynthesis in *Salmonella enterica* serovar Borreze. J Biol Chem 271:28581–28592. doi:10.1074/jbc.271.45.28581. 8910488

[16] Touchon M, Hoede C, Tenaillon O, Barbe V, Baeriswyl S, Bidet P, Bingen E, Bonacorsi S, Bouchier C, Bouvet O, Calteau A, Chiapello H, Clermont O, Cruveiller S, Danchin A, Diard M, Dossat C, Karoui ME, Frapy E, Garry L, Ghigo JM, Gilles AM, Johnson J, Le Bouguénec C, Lescat M, Mangenot S, Martinez-Jéhanne V, Matic I, Nassif X, Oztas S, Petit MA, Pichon C, Rouy Z, Ruf CS, Schneider D, Tourret J, Vacherie B, Vallenet D, Médigue C, Rocha EPC, Denamur E 2009 Organized genome dynamics in the *Escherichia coli* species results in highly diverse adaptive paths. PLoS Genet 5:e1000344. doi:10.1371/journal.pgen.1000344. 19165319 PMC2617782

[17] Magadum S, Banerjee U, Murugan P, Gangapur D, Ravikesavan R 2013 Gene duplication as a major force in evolution. J Genet 92:155–161. doi:10.1007/s12041-013-0212-8. 23640422

[18] Kliebenstein DJ 2008 A role for gene duplication and natural variation of gene expression in the evolution of metabolism. PLoS One 3:e1838. doi:10.1371/journal.pone.0001838. 18350173 PMC2263126

[19] Huerta-Cepas J, Szklarczyk D, Forslund K, Cook H, Heller D, Walter MC, Rattei T, Mende DR, Sunagawa S, Kuhn M, Jensen LJ, von Mering C, Bork P 2016 eggNOG 4.5: a hierarchical orthology framework with improved functional annotations for eukaryotic, prokaryotic and viral sequences. Nucleic Acids Res 44:D286–D293. doi:10.1093/nar/gkv1248. 26582926 PMC4702882

[20] Reeves PR, Cunneen MM, Liu B, Wang L 2013 Genetics and evolution of the *Salmonella* galactose-initiated set of o antigens. PLoS One 8:e69306. doi:10.1371/journal.pone.0069306. 23874940 PMC3715488

[21] Hong Y, Duda KA, Cunneen MM, Holst O, Reeves PR 2013 The WbaK acetyltransferase of *Salmonella enterica* group E gives insights into O antigen evolution. Microbiology 159:2316–2322. doi:10.1099/mic.0.069823-0. 24014662

[22] Richter M, Rosselló-Móra R 2009 Shifting the genomic gold standard for the prokaryotic species definition. Proc Natl Acad Sci U S A 106:19126–19131. doi:10.1073/pnas.0906412106. 19855009 PMC2776425

[23] Roach DJ, Burton JN, Lee C, Stackhouse B, Butler-Wu SM, Cookson BT, Shendure J, Salipante SJ 2015 A year of infection in the intensive care unit: prospective whole genome sequencing of bacterial clinical isolates reveals cryptic transmissions and novel microbiota. PLoS Genet 11:e1005413. doi:10.1371/journal.pgen.1005413. 26230489 PMC4521703

[24] DebRoy C, Fratamico PM, Yan X, Baranzoni G, Liu Y, Needleman DS, Tebbs R, O’Connell CD, Allred A, Swimley M, Mwangi M, Kapur V, Raygoza Garay JA, Roberts EL, Katani R 2016 Comparison of O-antigen gene clusters of all O-serogroups of *Escherichia coli* and proposal for adopting a new nomenclature for O-typing. PLoS One 11:e0147434. doi:10.1371/journal.pone.0147434. 26824864 PMC4732683

[25] Li P, Liu Q, Luo H, Liang K, Han Y, Roland KL, Curtiss R, 3rd, Kong Q 2018 Bivalent polysaccharides of Vi capsular and O9 O-antigen in attenuated *Salmonella* Typhimurium induce strong immune responses against these two antigens. NPJ Vaccines 3:1. doi:10.1038/s41541-017-0041-5. 29354293 PMC5760606

[26] Broadbent SE, Davies MR, van der Woude MW 2010 Phase variation controls expression of Salmonella lipopolysaccharide modification genes by a DNA methylation-dependent mechanism. Mol Microbiol 77:337–353. doi:10.1111/j.1365-2958.2010.07203.x. 20487280 PMC2909390

[27] Knirel YA, Prokhorov NS, Shashkov AS, Ovchinnikova OG, Zdorovenko EL, Liu B, Kostryukova ES, Larin AK, Golomidova AK, Letarov AV 2015 Variations in O-antigen biosynthesis and O-acetylation associated with altered phage sensitivity in *Escherichia coli* 4s. J Bacteriol 197:905–912. doi:10.1128/JB.02398-14. 25512310 PMC4325112

[28] Kim M, Ryu S 2012 Spontaneous and transient defence against bacteriophage by phase-variable glucosylation of O-antigen in *Salmonella enterica* serovar Typhimurium. Mol Microbiol 86:411–425. doi:10.1111/j.1365-2958.2012.08202.x. 22928771

[29] Kong Q, Yang J, Liu Q, Alamuri P, Roland KL, Curtiss R III. 2011 Effect of deletion of genes involved in lipopolysaccharide core and O-antigen synthesis on virulence and immunogenicity of *Salmonella enterica* serovar typhimurium. Infect Immun 79:4227–4239. doi:10.1128/IAI.05398-11. 21768282 PMC3187260

[30] Bogomolnaya LM, Santiviago CA, Yang H-J, Baumler AJ, Andrews-Polymenis HL 2008 “Form variation” of the O12 antigen is critical for persistence of *Salmonella* Typhimurium in the murine intestine. Mol Microbiol 70:1105–1119. doi:10.1111/j.1365-2958.2008.06461.x. 18826410

[31] Dlabac V, Trebichavský I, Reháková Z, Hofmanová B, Splíchal I, Cukrowska B 1997 Pathogenicity and protective effect of rough mutants of *Salmonella* species in germ-free piglets. Infect Immun 65:5238–5243. 9393821 10.1128/iai.65.12.5238-5243.1997 PMC175754

[32] Popoff MY, Le Minor L 1985 Expression of antigenic factor O:54 is associated with the presence of a plasmid in *Salmonella*. Ann Inst Pasteur Microbiol 136:169–179. doi:10.1016/S0769-2609(85)80042-9. 2417542

[33] Curd H, Liu D, Reeves PR 1998 Relationships among the O-antigen gene clusters of *Salmonella enterica* groups B, D1, D2, and D3. J Bacteriol 180:1002–1007. 9473060 10.1128/jb.180.4.1002-1007.1998 PMC106985

[34] Cota I, Sánchez-Romero MA, Hernández SB, Pucciarelli MG, García-Del Portillo F, Casadesús J 2015 Epigenetic control of *Salmonella enterica* O-antigen chain length: a tradeoff between virulence and bacteriophage resistance. PLoS Genet 11:e1005667. doi:10.1371/journal.pgen.1005667. 26583926 PMC4652898

[35] ChemAxon. Software solutions and services for chemistry and biology. ChemAxon.

[36] Ebrahim A, Lerman JA, Palsson BO, Hyduke DR 2013 COBRApy: COnstraints-Based Reconstruction and Analysis for Python. BMC Syst Biol 7:74. doi:10.1186/1752-0509-7-74. 23927696 PMC3751080

[37] Monk JM, Lloyd CJ, Brunk E, Mih N, Sastry A, King Z, Takeuchi R, Nomura W, Zhang Z, Mori H, Feist AM, Palsson BO 2017 iML1515, a knowledgebase that computes *Escherichia coli* traits. Nat Biotechnol 35:904–908. doi:10.1038/nbt.3956. 29020004 PMC6521705

[38] Charusanti P, Chauhan S, McAteer K, Lerman JA, Hyduke DR, Motin VL, Ansong C, Adkins JN, Palsson BO 2011 An experimentally-supported genome-scale metabolic network reconstruction for *Yersinia pestis* CO92. BMC Syst Biol 5:163. doi:10.1186/1752-0509-5-163. 21995956 PMC3220653

[39] Nogales J, Gudmundsson S, Duque E, Ramos JL, Palsson BO 2017 Expanding the computable reactome in pseudomonas putida reveals metabolic cycles providing robustness. bioRxiv

[40] Liao Y-C, Huang T-W, Chen F-C, Charusanti P, Hong JSJ, Chang H-Y, Tsai S-F, Palsson BO, Hsiung CA 2011 An experimentally validated genome-scale metabolic reconstruction of *Klebsiella pneumoniae* MGH 78578, iYL1228. J Bacteriol 193:1710–1717. doi:10.1128/JB.01218-10. 21296962 PMC3067640

[41] Kumar VS, Maranas CD 2009 GrowMatch: an automated method for reconciling in silico/*in vivo* growth predictions. PLoS Comput Biol 5:e1000308. doi:10.1371/journal.pcbi.1000308. 19282964 PMC2645679

[42] Orth JD, Thiele I, Palsson BØ 2010 What is flux balance analysis? Nat Biotechnol 28:245–248. doi:10.1038/nbt.1614. 20212490 PMC3108565

[43] Purdy ER Encyclopedia of global health. Center for Food Safety and Applied Nutrition.

[44] Timme RE, Pettengill JB, Allard MW, Strain E, Barrangou R, Wehnes C, Van Kessel JS, Karns JS, Musser SM, Brown EW 2013 Phylogenetic diversity of the enteric pathogen *Salmonella enterica* subsp. enterica inferred from genome-wide reference-free SNP characters. Genome Biol Evol 5:2109–2123. doi:10.1093/gbe/evt159. 24158624 PMC3845640

[45] Huang Y, Niu B, Gao Y, Fu L, Li W 2010 CD-HIT Suite: a web server for clustering and comparing biological sequences. Bioinformatics 26:680–682. doi:10.1093/bioinformatics/btq003. 20053844 PMC2828112

[46] Verma N, Reeves P 1989 Identification and sequence of *rfbS* and *rfbE*, which determine antigenic specificity of group A and group D salmonellae. J Bacteriol 171:5694–5701. doi:10.1128/jb.171.10.5694-5701.1989. 2793833 PMC210416

[47] Guan S, Clarke AJ, Whitfield C 2001 Functional analysis of the galactosyltransferases required for biosynthesis of D-galactan I, a component of the lipopolysaccharide O1 antigen of *Klebsiella pneumoniae*. J Bacteriol 183:3318–3327. doi:10.1128/JB.183.11.3318-3327.2001. 11344139 PMC99629

[48] Wattam AR, Davis JJ, Assaf R, Boisvert S, Brettin T, Bun C, Conrad N, Dietrich EM, Disz T, Gabbard JL, Gerdes S, Henry CS, Kenyon RW, Machi D, Mao C, Nordberg EK, Olsen GJ, Murphy-Olson DE, Olson R, Overbeek R, Parrello B, Pusch GD, Shukla M, Vonstein V, Warren A, Xia F, Yoo H, Stevens RL 2017 Improvements to PATRIC, the all-bacterial Bioinformatics Database and Analysis Resource Center. Nucleic Acids Res 45:D535–D542. doi:10.1093/nar/gkw1017. 27899627 PMC5210524

[49] Li D, Liu B, Chen M, Guo D, Guo X, Liu F, Feng L, Wang L 2010 A multiplex PCR method to detect 14 *Escherichia coli* serogroups associated with urinary tract infections. J Microbiol Methods 82:71–77. doi:10.1016/j.mimet.2010.04.008. 20434495

